# Supplementary material for: Taorong-type Baijiu starter: Analysis of fungal community and metabolic characteristics of middle-temperature Daqu and high-temperature Daqu
Source: PLoS One. 2022 Oct 4;17(10):e0274881. doi: 10.1371/journal.pone.0274881 (PMC9531834; doi:10.1371/journal.pone.0274881)
Supplement: Attachment — Submitted filename: Response to Reviewers.docx [file pone.0274881.s001.docx]

Dear Editor and Reviewers:

Thank you again for your letter and for the reviewers' comments concerning our manuscript. Those comments are all valuable and very helpful for revising and improving our paper, as well as the important guiding significance to our researches. We have studied comments carefully and have made correction which we hope meet with approval. The main corrections in the paper and the responds to the reviewer 's comments are as following:

Journal Requirements:

1. Please ensure that your manuscript meets PLOS ONE's style requirements, including those for file naming. The PLOS ONE style templates can be found at

https://journals.plos.org/plosone/s/file?id=wjVg/PLOSOne_formatting_sample_main_body.pdf and

https://journals.plos.org/plosone/s/file?id=ba62/PLOSOne_formatting_sample_title_authors_affiliations.pdf

It has been modified.

2. We note that the grant information you provided in the ‘Funding Information’ and ‘Financial Disclosure’ sections do not match.

When you resubmit, please ensure that you provide the correct grant numbers for the awards you received for your study in the ‘Funding Information’ section.

It has been modified.

3. Thank you for stating the following financial disclosure:

“Funding

This work was supported by the Key Technologies Research and Development Program of Henan Province of China (202102110130), Major Science and Technology Projects of Henan Province of China (181100211400), the Scientific Research Foundation for Docotors of Henan University of Animal Husbandry and Economy (2018HNUAHEDF011) and the Key Subject Projects of Henan University of Animal Husbandry and Economy(C3060020).”

Please state what role the funders took in the study. If the funders had no role, please state: "The funders had no role in study design, data collection and analysis, decision to publish, or preparation of the manuscript."

If this statement is not correct you must amend it as needed.

Please include this amended Role of Funder statement in your cover letter; we will change the online submission form on your behalf.

It has been modified. I have added after the funding “all awarded to YL. The funders had no role in study design, data collection and analysis, decision to publish, or preparation of the manuscript.”

4. Thank you for stating the following in the Acknowledgments Section of your manuscript:

“This work was supported by the Key Technologies Research and Development Program of Henan Province of China (202102110130), Major Science and Technology Projects of Henan Province of China (181100211400), the Scientific Research Foundation for Docotors of Henan University of Animal Husbandry and Economy (2018HNUAHEDF011) and the Key Subject Projects of Henan University of Animal Husbandry and Economy(C3060020).”

We note that you have provided additional information within the Acknowledgements Section that is not currently declared in your Funding Statement. Please note that funding information should not appear in the Acknowledgments section or other areas of your manuscript. We will only publish funding information present in the Funding Statement section of the online submission form.

Please remove any funding-related text from the manuscript and let us know how you would like to update your Funding Statement. Currently, your Funding Statement reads as follows:

“Funding

This work was supported by the Key Technologies Research and Development Program of Henan Province of China (202102110130), Major Science and Technology Projects of Henan Province of China (181100211400), the Scientific Research Foundation for Docotors of Henan University of Animal Husbandry and Economy (2018HNUAHEDF011) and the Key Subject Projects of Henan University of Animal Husbandry and Economy(C3060020). “

Please include your amended statements within your cover letter; we will change the online submission form on your behalf.

I have removed Funding in the manuscript. please show it elsewhere.

5. In your Data Availability statement, you have not specified where the minimal data set underlying the results described in your manuscript can be found. PLOS defines a study's minimal data set as the underlying data used to reach the conclusions drawn in the manuscript and any additional data required to replicate the reported study findings in their entirety. All PLOS journals require that the minimal data set be made fully available. For more information about our data policy, please see http://journals.plos.org/plosone/s/data-availability.

Upon re-submitting your revised manuscript, please upload your study’s minimal underlying data set as either Supporting Information files or to a stable, public repository and include the relevant URLs, DOIs, or accession numbers within your revised cover letter. For a list of acceptable repositories, please see http://journals.plos.org/plosone/s/data-availability#loc-recommended-repositories. Any potentially identifying patient information must be fully anonymized.

Important: If there are ethical or legal restrictions to sharing your data publicly, please explain these restrictions in detail. Please see our guidelines for more information on what we consider unacceptable restrictions to publicly sharing data: http://journals.plos.org/plosone/s/data-availability#loc-unacceptable-data-access-restrictions. Note that it is not acceptable for the authors to be the sole named individuals responsible for ensuring data access.

We will update your Data Availability statement to reflect the information you provide in your cover letter.

Relevant data has been uploaded to NCBI database，and the accession numbers is PRJNA861706.

6. Please ensure that you refer to Figure 1 in your text as, if accepted, production will need this reference to link the reader to the figure.

It has been modified.

7. Please review your reference list to ensure that it is complete and correct. If you have cited papers that have been retracted, please include the rationale for doing so in the manuscript text, or remove these references and replace them with relevant current references. Any changes to the reference list should be mentioned in the rebuttal letter that accompanies your revised manuscript. If you need to cite a retracted article, indicate the article’s retracted status in the References list and also include a citation and full reference for the retraction notice.

It has been modified.

Response to Reviewer #1:

Abstract

Line 24: In the results section The word largest is often overused in the text, its recommended the use of the most significant.

It has been modified.

Line 26: respectively, It appears that you have an unnecessary comma in a compound predicate. Consider removing it.

It has been modified.

Line 29: , and. Your sentence contains a series of three or more words, phrases, or clauses. Consider inserting a comma to separate the elements.

It has been modified.

Keywords

Line 35: , Fungal It appears that you have improperly spaced some punctuation. Consider adding a space.

It has been modified.

Introduction

Well done, only consider:

Line 37: , and. Your sentence contains a series of three or more words, phrases, or clauses. Consider inserting a comma to separate the elements.

It has been modified.

Line 53: fpr change by for

It has been modified.

Line 64: It seems that you have an unnecessary comma. Consider removing the comma.

It has been modified.

Materials and Methods

Line 95: iceboxes The word ice boxes seems to be miswritten. Consider replacing it.

It has been modified.

Line 97: It seems that semicolon use may be incorrect here. Consider using a comma instead of a semicolon

It has been modified.

Line 99: Consider adding a transition phrase to improve the flow of your paragraph. In addition, AMPure XP…

It has been modified.

Line 140: It appears that then may be unnecessary in this sentence. Consider removing it.

It has been modified.

Line 143: The noun phrase template seems to be missing a determiner before it. Consider adding an article.

It has been modified.

Line 149: Your sentence may be unclear or hard to follow. Consider rephrasing. (Option: The clean tags were clustered, and chimeric tags identified during this process were removed using UCHIME from USEARCH, leaving the remaining effective tags)

It has been modified.

Line 153: The phrase on the basis of may be wordy. Consider changing the wording (based on)

Results

It has been modified.

Line 161: The phrase the removal of may be wordy. Consider changing the wording. (removing)

It has been modified.

Line 171: Delete respectively.

It has been modified.

Line 222: Change: Monascus can produce various enzymes during the growth process, and their high enzymatic esterifying activity can catalyze the synthesis of acids and alcohols that are significant sources of fragrance in liquors

It has been modified.

Funding

Line 347: Doctors

It has been modified.

Response to Reviewer #2:

ABSTRACT

The full title is confusing, the Taorong-Type Daqu, it is liquor flavor and Daqu is a type or fermentation, it must be written in English which is the language of the article and improve writing of the title to link the objective with the liquor. Must be understood in English by all readers. Maybe Fermented liquor flavor Taorong type Daqu or something.

The same for the Short title

Clarify in methods molecular identification of microorganism communities and characterization of the volatile components of the fermentation liquor Taorong flavor type Daqu

Keywords, should not be words included in the title

Revised and improved the title, new title: Taorong-type Baijiu Starter: Analysis of Fungal Community and Metabolic Characteristics of Middle-Temperature Daqu and High-Temperature Daqu. Redefined keywords: Correlation, Baijiu starter, Flavor ingredient, Microbial community.

INTRODUCTION

Review writing errors and spaces in text

Unify the format of references in terms of spaces, score, etc.

The above notes have been modified.

METHODOLOGY

Clarify that a fermentation liquor that was used, is a micro-ecological product enriched with communities of microorganisms called Daqu Taorong- flavor or something and then name this Taorong-flavored Daqu. First explain, what were the temperatures medium and high

The medium-temperature Daqu (The incubation temperature is between 50°C and 60°C, and the maximum does not exceed 60°C) and high-temperature Daqu (The incubation temperature is above 60°C, and the maximum temperature can reach 70°C) were marked D-Z and E-G, respectively.

The subtitle Total DNA extraction and quantitative pcr…. it is suggested to change to molecular identification of microorganism communities of taorong-flavored daqu liquor fermentation by PCR system

It has been modified.

RESULTS

reference the table in the text

first goes the explanation, discussion of results and then the table

separate discussions from conclusions, discuss in the results

It has been modified.

We tried our best to improve the manuscript and made some changes in the manuscript. These changes will not influence the content and framework of the paper. We appreciate for Editor and Reviewers' warm work earnestly, and hope that the correction will meet with approval. If there are any shortcomings in the article, please tell me immediately, and I will seriously revise it again.Once again, thank you very much for your comments and suggestion.

Thank you and best regards.

Yours sincerely,

Yanbo Liu E-mail: yanboliu@hnuahe.edu.cn
